# ATP citrate lyase mediated cytosolic acetyl-CoA biosynthesis increases mevalonate production in *Saccharomyces cerevisiae*

**DOI:** 10.1186/s12934-016-0447-1

**Published:** 2016-03-03

**Authors:** Sarah Rodriguez, Charles M. Denby, T. Van Vu, Edward E. K. Baidoo, George Wang, Jay D. Keasling

**Affiliations:** Joint BioEnergy Institute, Emeryville, CA USA; Physical Biosciences Division, Lawrence Berkeley National Laboratory, Berkeley, CA USA; California Institute of Quantitative Biosciences (QB3), University of California, Berkeley, CA USA; Department of Bioengineering, University of California, Berkeley, CA USA; Department of Chemical and Biomolecular Engineering, University of California, Berkeley, CA USA

**Keywords:** ATP citrate lyase, Mevalonate pathway, *Saccharomyces cerevisiae*, Acetyl Coenzyme A, Metabolic engineering, Isoprenoid synthesis

## Abstract

**Background:**

With increasing concern about the environmental impact of a petroleum based economy, focus has shifted towards greener production strategies including metabolic engineering of microbes for the conversion of plant-based feedstocks to second generation biofuels and industrial chemicals.* Saccharomyces cerevisiae* is an attractive host for this purpose as it has been extensively engineered for production of various fuels and chemicals. Many of the target molecules are derived from the central metabolite and molecular building block, acetyl-CoA. To date, it has been difficult to engineer* S. cerevisiae* to continuously convert sugars present in biomass-based feedstocks to acetyl-CoA derived products due to intrinsic physiological constraints—in respiring cells, the precursor pyruvate is directed away from the endogenous cytosolic acetyl-CoA biosynthesis pathway towards the mitochondria, and in fermenting cells pyruvate is directed towards the byproduct ethanol. In this study we incorporated an alternative mode of acetyl-CoA biosynthesis mediated by ATP citrate lyase (ACL) that may obviate such constraints.

**Results:**

We characterized the activity of several heterologously expressed ACLs in crude cell lysates, and found that ACL from *Aspergillus nidulans* demonstrated the highest activity. We employed a push/pull strategy to shunt citrate towards ACL by deletion of the mitochondrial NAD^+^-dependent isocitrate dehydrogenase (IDH1) and engineering higher flux through the upper mevalonate pathway. We demonstrated that combining the two modifications increases accumulation of mevalonate pathway intermediates, and that both modifications are required to substantially increase production. Finally, we incorporated a block strategy by replacing the native ERG12 (mevalonate kinase) promoter with the copper-repressible CTR3 promoter to maximize accumulation of the commercially important molecule mevalonate.

**Conclusion:**

By combining the push/pull/block strategies, we significantly improved mevalonate production. We anticipate that this strategy can be used to improve the efficiency with which industrial strains of* S. cerevisiae* convert feedstocks to acetyl-CoA derived fuels and chemicals.

**Electronic supplementary material:**

The online version of this article (doi:10.1186/s12934-016-0447-1) contains supplementary material, which is available to authorized users.

## Background

Increasing environmental concerns about the sustainability of producing petroleum-derived chemicals has led to increased interest in alternative means of production. Metabolic engineering of microorganisms to convert biomass and organic waste to chemicals that are ordinarily derived from petroleum represents a more sustainable production strategy. The yeast *Saccharomyces cerevisiae* is a commonly used microbial cell factory for metabolic engineering [2, 3, 28] as it is tolerant of industrial conditions, is extraordinarily well-characterized, and offers tools for genetic engineering [19, 20, 29]. While an immense amount of progress has been made, there remains room to improve the efficiency with which engineered microbes produce chemicals from feedstocks.

Acetyl-CoA is a key molecule in central carbon metabolism [23], as it is required for basic cellular functions such as energy metabolism, lipid metabolism, and amino acid metabolism. Acetyl-CoA also serves as a precursor for the biosynthesis of many industrial chemicals including lipids (dietary supplements and biodiesels), polyketides (antibiotics and anticancer drugs), polyhydroxyalkanoates (biodegradable polymers), and isoprenoids (flavors and fragrances, biodiesels, anti-microbials and anti-cancer drugs, rubber, cosmetic additives and vitamins). In addition, the isoprenoid pathway intermediate mevalonate has been cited for its use as a precursor for biobased production of β-methyl-δ-valerolactone, which can be transformed into a rubbery polymer [42].

In *S. cerevisiae*, cytoplasmic acetyl-CoA is endogenously produced from pyruvate through three metabolic steps called the pyruvate dehydrogenase (PDH) bypass. However, due to the nature of the endogenous regulatory metabolism, production of acetyl-CoA derived metabolites via the native cytoplasmic PDH bypass is constrained: when grown at high glucose concentrations a significant portion of glucose is converted to reduced byproducts such as ethanol [41], whereas at low glucose concentrations the bulk of pyruvate is transported to the mitochondria [32].

Once pyruvate enters the mitochondrion it can be converted to acetyl-CoA by the PDH complex, however, this mitochondrial acetyl-CoA pool is not available to cytoplasmic biosynthesis pathways.

**Fig. 1:**
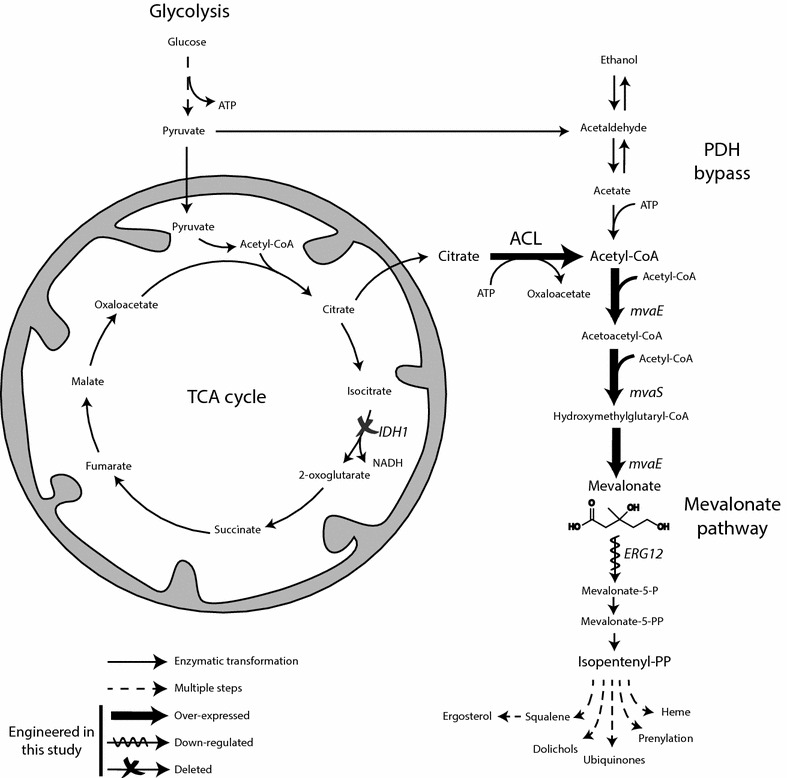
Diagram of *S. cerevisiae* metabolic pathways relevant to this study. Engineered enzymatic steps of native yeast metabolism are *IDH1*, isocitrate dehydrogenase 1 and *ERG12*, mevalonate kinase. Non-native engineered enzymatic steps are ACL, ATP citrate lyase of *Aspergillus nidulans*; *mvaE* acetoacetyl-CoA synthase and HMG-reductase of *Enterococcus faecalis*; *mvaS*, acetoacetyl-CoA thiolase of *Enterococcus faecalis*

Although a transport mechanism for shuttling acetyl-CoA from the mitochondrion to the cytoplasm does not exist in *S. cerevisiae*, many other organisms shuttle acetyl-CoA through the intermediate citrate. This is typified by oleaginous yeast species, which are defined by their ability to accumulate high levels of cytoplasmic acetyl-CoA derived triacylglycerides in nitrogen-limiting conditions [33, 34]. At the genomic level, a key characteristic of oleaginous yeasts is that they contain genes encoding the ATP citrate lyase (ACL), whereas non-oleaginous yeasts such as *S. cerevisiae* do not [1]. It has been demonstrated that in low nitrogen conditions, inhibition of isocitrate dehydrogenase (ICDH) leads to citrate accumulation, cytosolic transport, and cleavage by ACL thus generating cytoplasmic acetyl-CoA [9, 10] (Fig. 1).

In this study, we reconstituted the salient features of the ACL pathway in *S. cerevisiae* through genetic engineering and media optimization. As a proof of principle, we demonstrate that this alternative mode of metabolism can be used to significantly increase the yield of the acetyl-CoA derived product mevalonate. *E. coli* has also been engineered to produce mevalonate as a final product [42]. While the mevalonate pathway in *S. cerevisiae* has been extensively engineered to produce various isoprenoid products, this is the first study to our knowledge, to produce mevalonate as a final product.

## Methods

### Media, cultivation, and transformation

For strain construction, pre-cultures were grown with 5 mL of Yeast extract + Peptone + Dextrose (YPD) medium in glass test tubes with shaking at 200 rpm. After 18 h of growth, pre-cultures were used to inoculate 50-mL cultures in 250-mL Erlenmeyer flasks. After ~6 h of growth, strains were transformed by the lithium acetate method [14]. For construction of *IDH1::HygB*, the Hygromycin B resistance cassette was amplified from pUG32 [15] with primers containing 40 base pairs corresponding to the chromosomal sequence immediately 5′ and 3′ of the *IDH1* locus. For construction of strains containing integrated heterologous gene sequences, the integration fragments were amplified from their corresponding plasmids (Additional file 1: Table S1). Each integration fragment contained the heterologous genes and either an auxotrophic complementation or drug resistance cassette, with flanking 750 base pairs corresponding to the site of chromosomal integration. To select for DNA transformants containing auxotrophic complementation cassettes, cells were plated on standard dropout medium (Sunrise, San Diego), and for DNA transformants containing drug resistance cassettes, cells were plated on YPD, grown for 24 h, and then replica plated to YPD supplemented with 200 μg/L Geneticin (Sigma, cat. no. A1720) or Hygromycin B (Sigma, cat. no. H7772).

For the ACL activity assay, single colonies were used to inoculate 5 mL Yeast extract + Peptone (YP) + 2 % galactose liquid cultures. Cultures were then diluted into fresh medium to an OD of 0.05, and grown for 24 h.

For metabolomics and production experiments, strains were grown at 30 °C with shaking at 200 rpm in a base medium composed of 6.7 g/L Yeast Nitrogen Base (YNB) without amino acids, without ammonium sulfate (Difco), and 0.7 g/L Complete Supplemented Media (CSM) amino acid mixture (Sunrise, San Diego). Media were supplemented with additional components as specified below. For metabolomics quantification, cultures were grown in the base medium supplemented with 2 % raffinose and 0.1 % dextrose. Five milliliter pre-cultures were inoculated from single colonies and grown for 36 h in test tubes. Production cultures (50 mL) supplemented with 2 % galactose were inoculated to an initial OD_600_ of 0.05 in 250-mL Erlenmeyer flasks. Low nitrogen cultures were supplemented with 7 mg/L ammonium sulfate, and standard nitrogen cultures were supplemented with 1 g/L ammonium sulfate.

For experiments ‘Increasing pull on cytoplasmic acetyl-CoA towards the mevalonate pathway,’ 10-mL pre-cultures supplemented with 2 % galactose were grown for 36 h in test tubes. These cultures were used to inoculate an additional 10-mL pre-culture to an initial OD_600_ of 0.2, which was grown for 6 h. The resulting cultures were centrifuged at 3000×*g* and washed once with fresh base medium. A fraction of the resulting cell suspension was centrifuged and resuspended in 10 mL of fresh base medium in test tubes so as to reach an initial OD_600_ of 0.5, and then 2 × 7 mm silicone elastomer galactose discs (Kuhner) were added.

### Gene synthesis

Coding sequences for *ACLa* and *ACLb* from *Aspergillus nidulans* were obtained from the Aspergillus Comparative Database (http://www.broadinstitute.org/annotation/genome/aspergillus_group). Both genes were codon optimized for *S. cerevisiae* and synthesized by Genscript (Piscataway, New Jersey). The genes were cloned into *pADS*-*AMO*-*CPR* [35] to generate JBEI-7134. Additional ACL coding sequences were obtained at the following locations: the *Rhodosporidiumtoruloides* gene was obtained from the Genomics Web Portal (http://crdd.osdd.net/raghava/genomesrs/); the *Yarrowia lipolytica* genes were obtained from the Genelovures Yeast Genomes Database (http://genolevures.org/yali.html); the *Lipomyces starkeyi* genes were obtained from the JGI Genome Portal (http://genome.jgi.doe.gov/Lipst1_1/Lipst1_1.home.html); and the *Mus musculus* gene was obtained from the NCBI Gene Database (http://www.ncbi.nlm.nih.gov/gene/104112). Coding sequences were optimized for expression in *S. cerevisiae* using the IDT codon optimization tool, and were cloned into the pESC-*LEU2d* (strain Keasling-1951) parent plasmid by JGI to generate JBEI-10648, and JBEI-10641–10644 (see Table 1).

**Table 1:** Strains and plasmids

Strain	Genotype	Reference
BY4741	*MATa his3Δ1 leu2Δ0 met15Δ0 ura3Δ0*	Euroscarf
JBEI-10683	BY4741 *ura3:*:P_GAL10_-*AnACLb*-P_GAL1_-*AnACLa*-*kanMX4*	This study
JBEI-10684	BY4741 *ura3::*P_GAL1_-*RtACL*-*kanMX4*	This study
JBEI-10685	BY4741 *ura3::*P_GAL1_-*MmACL*-*kanMX4*	This study
JBEI-10686	BY4741 *ura3:*:P_GAL10_-*YlACLb*-P_GAL1_-*YlACLa*-*kanMX4*	This study
JBEI-10687	BY4741 *ura3:*:P_GAL10_-*LsACLb*-P_GAL1_-*LsACLa*-*kanMX4*	This study
JBEI-10688	BY4741 *idh1::kanMX4*	Euroscarf
JBEI-10689	BY4741 *ura3:*:P_GAL10_-*AnACLb*-P_GAL1_-*AnACLa*-*kanMX4 idh1::hphMX4*	This study
JBEI-10569	BY4741 *trp1::EfMvaE*-*EfMvaS*–*SpHIS5*	This study
JBEI-10690	BY4741 *ura3:*:P_GAL10_-*AnACLb*-P_GAL1_-*AnACLa*-*kanMX4 trp1::EfMvaE*-*EfMvaS*–*SpHIS5*	This study
JBEI-10691	BY4741 *ura3:*:P_GAL10_-*AnACLb*-P_GAL1_-*AnACLa*-*kanMX4 idh1::hphMX4* *trp1::EfMvaE*-*EfMvaS*–*SpHIS5*	This study
JBEI-10692	BY4741 *ura3:*:P_GAL10_-*AnACLb*-P_GAL1_-*AnACLa*-*kanMX4 idh1::ylIDH*–*CaURA3* *trp1::EfMvaE*-*EfMvaS*–*SpHIS5*	This study
JBEI-10693	BY4741 *ura3:*:P_GAL10_-*AnACLb*-P_GAL1_-*AnACLa*-*kanMX4 idh1::hphMX4 ERG12::KlLeu2*–P_CTR3_-*ERG12*	This study
JBEI-10694	BY4741 *ura3:*:P_GAL10_-*AnACLb*-P_GAL1_-*AnACLa*-*kanMX4 ERG12::KlLeu2*–P_CTR3_-*ERG12*	This study

Yeast strains used in this study

*MvaE* was codon optimized *for S. cerevisiae* and synthesized by Genscript (Piscataway, New Jersey). The coding sequences were obtained from NCBI cataloged under accession: AF290092.1 GI: 9937382. *MvaS* was amplified directly from the *Enterococcus faecalis* genome, with primers designed to amplify from the nucleotide sequence obtained from the same NCBI accession number.

### Plasmid construction

All strains (Table 1), expression plasmids (Table 2), additional plasmids used for constructing strains and expression plasmids (Additional file 1: Table S1), and the corresponding sequence files are described in the JBEI Public Registry (https://public-registry.jbei.org/) [16], and are available upon request. All plasmids built in this study used for strain and expression plasmid construction were constructed using Gibson assembly [13] or Yeast assembly [27]. Plasmids were designed using Device Editor bioCAD software [4], and assembly primers were generated with j5 DNA assembly design automation software [18] using the default settings. PCR amplification was performed using Prime STAR GXL DNA polymerase using the manufacturer’s instructions (Takara). Assemblies were performed using Gibson assembly master mix (New England Biolabs), and were transformed into DH10b competent cells for propagation. Plasmid DNA was purified using a QIAprep Spin Miniprep Kit (QIAGEN), and plasmids were sequenced with ~2X coverage (Quintara). DNA sequences derived from *S. cerevisiae* were amplified from genomic DNA prepared using a modified Miniprep protocol: 1 mL yeast cell culture in YPD medium was centrifuged in a screw cap tube (3000×*g*) and resuspended in buffer P1 (from Qiagen kit). Cells were lysed by adding glass beads and shaking in a benchtop homogenizer/bead beating instrument (FastPrep-24, MP Biomedicals) for ~1 min. Resulting suspension was used for remaining steps in Qiagen Miniprep protocol. Details of construction for each plasmid are as follows:

**Table 2:** Expression plasmids used in this study

Plasmid name	JBEI registry number	Description	Reference
pESC-LEU	JBEI-10738	Yeast episomal plasmid with 2μ-origin and *LEU2* selectable marker	Stratagene, La Jolla, CA
pACLab	JBEI-7134	pESC-P_GAL10_-*AnACLb*-P_GAL1_-*AnACLa*-*LEU2d*	This study
pESC-URA	JBEI-10737	Yeast episomal plasmid with 2μ-origin and *URA3* selectable marker	Stratagene, La Jolla, CA
pMvaES	JBEI-10632	pESC-P_GAL10_-*EfMvaE*-P_GAL1_-*EfMvaS*-*URA3*	This study
pYlIDH	JBEI-10650	pESC-P_GAL10_-*YlIDH1*-P_GAL1_-*YlIDH2*-*Leu2d*	This study

JBEI-7134 (pACLab). Two multi-copy plasmids, pRS426 [5] and pESC-URA (Stratagene, La Jolla), were previously combined to form plasmid *pADS*-*AMO*-*CPR* [35]. This plasmid contained genes encoding amorphadiene synthase (*ADS*), amorphadiene oxidase (*AMO*) and its redox partner cytochrome P450 reductase (*CPR*). We removed the AMO and CPR genes and replaced them with *ACLa* and *ACLb* genes, respectively, to form p*ESC*-P_*GAL1*_*ACLa*-P_*GAL*10_*ACLb*-P_*GAL*1_-*ADS*-*LEU2d* (JBEI-3271) plasmid. Then *ADS* was removed by yeast homologous recombination. Phosphorylated primers, with 20 overhanging base pairs and matching ends homologous to the promoter or terminator regions surrounding *ADS*, were designed to amplify the p*ESC*-P_*GAL1*_*ACLa*-P_*GAL*10_*ACLb*-P_*GAL*1_-*ADS*-*LEU2d* vector regions surrounding the *ADS* gene by PCR. The PCR product was transformed into yeast and colonies were screened for the removal of *ADS* by PCR. The resulting construct was purified using a modified Miniprep protocol (described above) and transformed into DH10b competent cells for propagation.

JBEI-10632 (pMvaES). This plasmid was constructed from Keasling-2159 by replacing *AMO* with *mvaE* and *CPR* with *mvaS* using standard yeast homologous recombination. Replacement of the *LEU2d* marker for the *URA3* marker was performed by cutting the plasmid once within the *LEU2d* marker, treatment of the plasmid with phosphatase, and co-transformation of the treated cut plasmid with PCR product of the *URA3* cassette, with flanking ends homologous to the site of integration within the vector.

JBEI-10650. DNA from *Y. Lipolytica* was prepared using the modified Mini prep protocol described above. The p*ESC*-*LEU2d* backbone, the *Y.l.IDH1* and P_*GAL1/*10_ fragments were amplified for Gibson assembly-based construction as described above. The genomic *Y.l.IDH2* gene contains an intron 24 base pairs 3′ from the start of the coding sequence. In order to remove the intron, the intron-less coding sequence was broken into two parts. The first part was amplified from a 500-bp gBlock (IDT), while the second part was amplified from genomic DNA.

### ACL Activity assay

Cell lysates were prepared as follows: 20 OD units of three biological replicate cultures were centrifuged at 3300×*g* for 10 min. Cells were washed in 2 mL of 0.1 M Tris buffer, pH 8.7 supplemented with 10 µL protease inhibitor (Sigma cat. no. P8215) and then resuspended in 500 µL of the same buffer. 500 µL of glass beads (USA scientific) were added, and cells were broken using a benchtop homogenizer/bead beating instrument (FastPrep-24, MP Biomedicals). Cells were shaken 5 × 20 s intervals, separated by one-minute intervals on ice. Beads and cellular debris were centrifuged at 10,000×*g* for 2 min at 4 °C. 300 µL of supernatant were collected as the cell lysate.

ACL activity assays were performed as described previously [26] with minor modifications. Briefly, 20 µL of cell lysate was added to a mixture composed of 100 mM Tris–HCl (pH 8.4), 20 mM sodium citrate, 5 u/ml malate dehydrogenase (Sigma), 10 mM MgCl_2_, 10 mM DTT, 0.15 μM NADH, 0.3 mM Coenzyme A, and 5 mM ATP. Reactions were initiated by adding cell lysate and the decrease in absorption at 340 nm was monitored every 15 s using a SPMAX plate reader set at 30 °C. Lysate activities were calculated as the initial rate of the decrease in absorption at 340 nm. Initial rates were calculated as the slope of a general linearized model for the first 10 min of the reaction. For the ACL-containing strains, activities were reported as the absolute initial rate divided by the initial rate of the parent strain, then divided by total mg protein in each sample as measured by Pierce BCA protein assay kit (Life Technologies).

### Citric acid cycle metabolite analysis

Metabolite extraction was performed as previously demonstrated by [38], modified for *S. cerevisiae* by use of bead-beating for cell lysis of five OD units of culture. Tri-carboxylic acid (TCA) cycle intermediates were analyzed by liquid chromatography and mass spectrometry (LC–MS). Chemical standards were made up to 200 µM, as the stock solution, in methanol–water (50:50, v/v). The separation of the TCA cycle intermediates was conducted on a ZIC-pHILIC column (150 mm length, 4.6-mm internal diameter, and 5-µm particle size; from Merck SeQuant, and distributed via The Nest Group, Inc., MA., USA) using an Agilent Technologies 1200 Series HPLC system (Agilent Technologies, CA, USA). The sample injection volume was 3 µL. The temperature of the sample tray was maintained at 4 °C using an Agilent FC/ALS Thermostat. The column compartment was set to 40 °C. The mobile phase was composed of (A) 10 mM ammonium carbonate and 0.5 % ammonium hydroxide in acetonitrile–water (2:8, v/v) and (B) 10 mM ammonium carbonate and 0.5 % ammonium hydroxide in acetonitrile–water (8:2, v/v). TCA cycle intermediates were eluted isocratically with a mobile phase composition of 33 % mobile phase A and 67 % of mobile phase B. A flow rate of 0.45 mL/min was used. The HPLC system was coupled to an Agilent Technologies 6210 time-of-flight mass spectrometer (LC-TOF MS) by a 1/6 post-column split. Contact between both instrument set-ups was established using a LAN card in order to trigger the MS into operation upon initiation of a run cycle from the MassHunter workstation (Agilent Technologies, CA, USA). Electrospray ionization (ESI) was conducted in the negative ion mode and a capillary voltage of—3500 V was used. MS experiments were carried out in full scan mode, at 0.86 spectra/second for the detection of [M–H]/Z. Prior to LC-TOF MS analysis, the TOF MS was calibrated via an ESI-L-low concentration tuning mix (Agilent Technologies, CA, USA). Data acquisition and processing were performed by the MassHunter software package. The instrument was tuned for a range of 50–1700 m/z—were quantified via eight-point calibration curve ands ranging from 625 nM to 200 µM. The R^2^ coefficients for the calibration curves were ≥0.99.

### CoA metabolite analysis

For CoA metabolite analysis, a method previously established was adapted from [7]. Briefly, 20 OD units of each culture were pelleted (6000 rpm, 5 min, 4 °C). The supernatant was aspirated, and the cells were suspended in 1 mL of 10 % TCA containing crotonyl-CoA (10 μM) as an internal standard. The cells were bead-beaten for a total of 5 min (intervals of 20 s of beating followed by 20 s on ice). The supernatant was collected and neutralized with 2 × volume of 1 M octylamine. Samples were then filtered and the neutralized TCA extract was analyzed via LC–MS using electrospray ionization. The LC conditions used were adapted from [31].

### Extracellular metabolites and organic acids detection

Glucose, galactose, acetate, ethanol and glycerol were separated by HPLC and detected by RID and DAD detectors. One mL of cell culture was transferred and centrifuged at 18,000×*g* for 5 min. The supernatant was then filtered using a Costar^®^ Spin-X^®^ Centrifuge Tube Filters, 0.22-µm pore and applied to an Agilent 1100 series HPLC equipped with an Agilent 1200 series auto-sampler, an Aminex HPX-87H ion exchange column (Biorad), and an Agilent 1200 series DAD and RID detectors. Metabolites were separated using 4 mM H_2_SO_4_ aqueous solution with a flow rate of 0.6 ml/min at 50 °C. Galactose consumption was calculated as the concentration of galactose remaining in prepared medium with cultured strains, subtracted from the concentration of galactose in the control culture of prepared media without cells.

### Mevalonate and squalene quantification

A detailed description of mevalonate and squalene quantification was recently described [36]. In brief, mevalonate was derivatized to mevalonolactone by mixing 200 µL cell culture with 50 µL 2 M HCl and vortexing for 15 min. Mevalonolactone was extracted into ethyl acetate by adding 250 µL ethyl acetate containing 10 μg/mL caryophyllene (internal standard) and vortexing for 5 min. Samples were centrifuged at 3000×*g* for 5 min and 100 µL of the organic phase was removed and transferred to glass GC vials for GCMS analysis. 1 μL of sample was injected (splitless), by using He as the carrier gas onto a CycloSil-B column (Agilent, 30-m length, 0.25-mm inner diameter (i. d.), 0.25-μm film thickness, cat. no. 112–6632) using an Agilent GC system 6890 series GCMS with Agilent mass selective detector 5973 network. The carrier gas was held at a constant flow rate of 1.0 mL/minutes. After each sample injection, the oven temperature was held at 90 °C for 1 min, followed by a ramp of 30 °C/minute to a final temperature of 250 °C, and then held at 250 °C for 2 min. Solvent delay was set to 3.5 min, EMV mode was set to a gain factor of 1, and the MS instrument was set to SIM for acquisition, monitoring *m/z* ions 58 and 71 (mevalonolactone ions), along with 189 and 204 (caryophyllene internal standard ions). Peak areas for mevalonolactone and caryophyllene were quantified using MSD Productivity ChemStation software (Agilent), and relative mevalonolactone levels were calculated as the quotient of mevalonolactone and caryophyllene. For absolute quantification, sample values were fit to a generalized linear model generated from mevalonolactone standards.

Squalene was extracted by resuspending cells from 2 mL culture in 0.4 mL of the alcoholic KOH solution containing 10 μg/mL cholesterol, and boiling for 5 min. Squalene was extracted from the alcoholic solution by adding 0.4 mL of dodecane and vortexing for 5 min. Samples were centrifuged at 3000×*g* for 5 min and 100 µL of the organic phase was removed to glass GC vials. GCMS analysis and quantification were as described above with the following modifications: samples were injected onto a DB-5MS column (Agilent); after each sample injection, the oven temperature was held at 80 °C for 1 min, followed by a ramp of 20 °C/min to 280 °C, and then held for 15 min at 280 °C and a ramp to 300 °C at a rate of 20 °C/min with a final hold at 300 °C for 2 min.; solvent delay was set to 10 min, EMV mode was set to a gain factor of 1, the mass spectrometer was set to SIM acquisition mode, monitoring *m*/*z* ions 218, 386 and 396, and the temperatures of the quadrupole and the ion source were set to 200 and 300 °C, respectively.

## Results

### Activity of ACLs from different sources

**Fig. 2:**
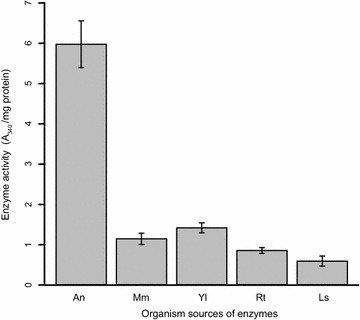
Activity measurements of heterologously expressed ACL from various organisms. Organisms are: An, *Aspergillus nidulans*; Mm, *Mus musculus*; Yl, *Yarrowia lipolytica*; Rt, *Rhodosporidium toruloides*; Ls, *Lipomyces starkeyii*. Respective strains are: An, JBEI-10683; Mm, JBEI-10685; Yl, JBEI-10686; Rt, JBEI-10684; Ls, JBEI-10687

Previous work has demonstrated that incorporating ACL from various organisms into *S. cerevisiae* improves production of acetyl-CoA derived products [11, 24, 40]. We synthesized yeast codon optimized ACL genes from five different sources—four from the fungi *A. nidulans*, *R.toruloides*, *Y. lipolytica*, and *L. starkeyii*, and one from the house mouse *Mus musculus*. In order to compare the activity of the heterologously expressed ACLs, we integrated each of the sequences into the *S. cerevisiae* genome, and measured their activity in crude cell lysates using an NADH-dependent coupled assay (Fig. 2). We observed a range of activity levels among the cell lysates. ACLs from *R.toruloides* and *L. starkeyi* displayed no detectable activity, ACLs from *Mus musculus* and *Y. lipolitica* displayed activity above background, consistent with previous publications [11, 24, 40]. The ACL from *A. nidulans* exhibited the highest activity by approximately an order of magnitude. Because the ACL from *A. nidulans* exhibited by far the highest activity, we decided to focus on this enzyme to further characterize expression of this ACL in *S. cerevisiae*.

### Engineering increased intracellular flux through ACL

**Fig. 3:**
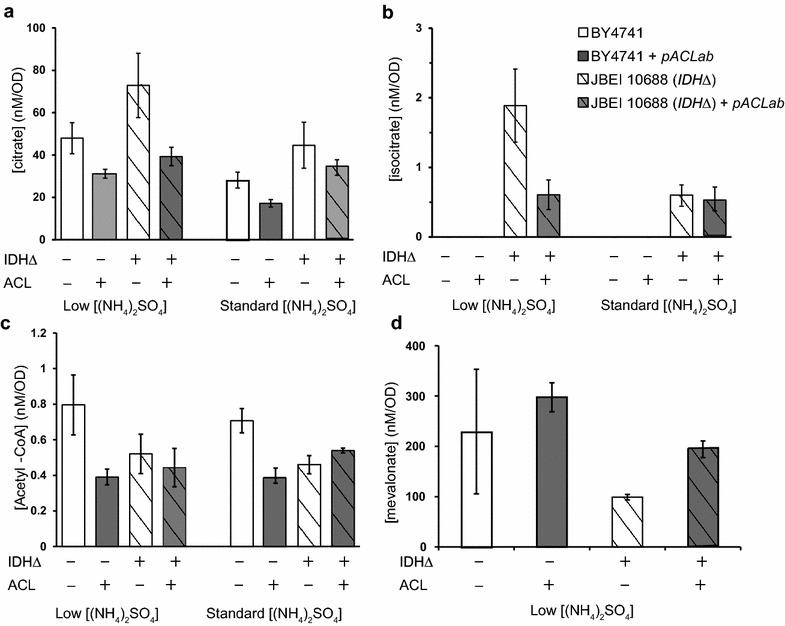
Intracellular concentrations of metabolites relevant to ACL activity as a function of nitrogen availability. Citrate (**a**), isocitrate (**b**), acetyl-CoA (**c**), mevalonate (**d**)

In *S. cerevisiae* citrate is primarily produced in the mitochondrion, where it is consumed by the citric acid cycle. A fraction of the mitochondrial citrate is transported to the cytoplasm by the citrate transporter CTP1 in exchange for malate [21]. The cytoplasmic citrate may then be consumed by ACL for cytoplasmic acetyl-CoA production. To determine whether *A. nidulans* ACL is active in vivo, and whether enough intracellular citrate is available for increased acetyl-CoA production, we measured the intracellular concentrations of citrate and acetyl-CoA in a strain containing *A. nidulans* ACL on a high copy plasmid. These strains were grown in both standard (i.e., supplemented to 1 g/L ammonium sulfate) and nitrogen limited conditions, as previous studies have reported higher activity of the mitochondrial citrate synthase in cells grown with low nitrogen concentration [22]. Citrate levels decreased to a similar extent in both conditions when ACL was expressed (39 % standard nitrogen; 35 % low nitrogen), suggesting that heterologously expressed ACL actively consumed citrate irrespective of nitrogen concentration (Fig. 3a). However, we indeed observed higher absolute levels of citrate in nitrogen-limited conditions when compared with the wild-type strain grown in medium with standard nitrogen supplementation (70 % increase) (Fig. 3a). The increase in total intracellular citrate concentrations associated with growth in limited nitrogen medium prompted us to adopt this growth condition for subsequent engineering experiments.

Surprisingly, the total cellular acetyl-CoA concentration decreased in both limited and standard nitrogen conditions with the expression of ACL (Fig. 3c). Because there are myriad cellular pathways that consume acetyl-CoA [12], we reasoned that total acetyl-CoA levels may not reflect flux through ACL. Consistently, several previous studies have demonstrated that total cellular acetyl-CoA concentrations between strains do not correlate with production of acetyl-CoA derived products [31, 39].

One of the major sinks for cytoplasmic acetyl-CoA in *S. cerevisiae* is the isoprenoid pathway, which leads to the biosynthesis of ubiquinone, prenylated proteins, and sterols (Fig. 1). Previous work has shown that strains engineered for isoprenoid production exhibit accumulation of the pathway intermediate mevalonate [30, 36]. We observed an increase in mevalonate suggesting that ACL expression may increase flux toward the mevalonate pathway, however the effect was modest (30 %) (Fig. 3c). We reasoned that the limited increase in flux to acetyl-CoA-dependent pathways was likely due to insufficient citrate supply.

In order to increase citrate supply to ACL, we attempted to further mimic the lipid accumulation phase of oleaginous yeast central carbon metabolism, by shunting the citrate destined for the TCA cycle to the cytoplasm. In oleaginous yeast, the major regulatory mechanism controlling the shift of citrate flux from the TCA cycle to ACL is mediated by inhibition of mitochondrial ICDH [9, 10]. *S. cerevisiae* contains both NADP^+^- and NAD^+^-dependent mitochondrial ICDHs. In a wild-type background the NAD^+^-dependent mitochondrial ICDH mediates the bulk of the cellular flux [6]. It has been shown previously that deletion of *IDH1*, which encodes the regulatory subunit of NAD^+^-dependent mitochondrial ICDH, results in increased citrate concentration [25], suggesting that *IDH1Δ* could effectively shunt citrate from the TCA cycle to the cytoplasm. This prompted us to measure the effect of *IDH1Δ* and ACL overexpression on the levels of other key central carbon metabolites.

Changes in metabolite concentrations in strains with *IDH1Δ* and ACL expression strongly suggest that we successfully reconstituted this key aspect of oleaginous yeast metabolism. First, isocitrate was undetectable in *IDH1*^+^ strains, but was clearly detectable in the *IDH1Δ* background, indicating that flux through ICDH was effectively blocked (Fig. 3b). Second, while citrate and isocitrate concentrations increased in the *IDH1Δ* background, both decreased dramatically when ACL was expressed, indicating that a substantial fraction of cellular citrate was transported from the mitochondrion to the cytoplasm and consumed by ACL (46 % decrease in citrate concentrations with ACL expression) (Fig. 3b, c). Third, while the mevalonate concentration decreased in the *IDH1Δ* background, it increased when ACL was expressed (96 % increase) (Fig. 3d), suggesting that shunting citrate from the TCA cycle may allow additional acetyl-CoA produced by ACL to be directed to acetyl-CoA dependent biosynthesis pathways.

Other key metabolites of the TCA cycle and glyoxylate shunt were measured (Additional file 2: Figure S1). *IDH1****∆***-modified strains demonstrate fold increases of intracellular concentrations of malate, fumarate and succinate by 2.5, 2.2, and 1.8, respectively, as compared to the wild type strain (Additional file 2: Figure S1). Interestingly, these metabolites are key products of the glyoxylate cycle, and large increases in intracellular concentrations of malate, fumarate, and succinate in *IDH1∆* strains may be indicative of utilization of the glyoxylate cycle. Notably, 2-oxoglutarate was also found to significantly increase (205 % increase) with ACL expression, as compared to the *IDH∆* background without ACL (Additional file 2: Figure S1). We speculate that the expression of ACL leads to down stream effects which replenish the mitochondria with 2-oxoglutarate for further oxidative phosphorylation.

### Increasing pull on cytoplasmic acetyl-CoA towards the mevalonate pathway

We next set out to test whether our strategy could be used to improve biosynthesis of acetyl-CoA derived products in a strain engineered for greater pull towards the mevalonate pathway. In particular we focused on production of the acetyl-CoA derived pathway intermediate mevalonate, because of its potential use as a precursor for biobased production of β-methyl-δ-valerolactone [42]. Acetyl-CoA flux was directed to the mevalonate pathway by integrating two genes from *Enterococcus faecalis, mvaE* and *mvaS,* that together encode the first three steps of the mevalonate pathway [17, 43, 44] (Fig. 1). Functional expression of these genes has not previously been demonstrated in *S. cerevisiae* to our knowledge.

**Fig. 4:**
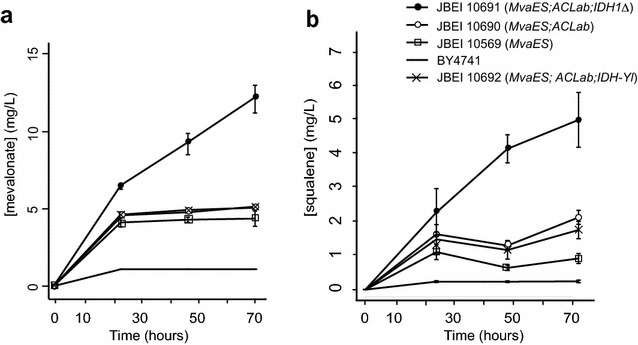
Time course of mevalonate production (**a**) and cellular squalene concentrations (**b**) in engineered strains. Strains are: No symbols represent the unengineered parent strain BY4741; Empty squares represent BY4741 with MvaES genes expressed, JBEI 10569; Emtpy circles represent BY4741 with MvaES and ACLab genes expressed, JBEI 10690; Filled circles represent BY4741 with MvaES and ACLab genes expressed, and the IDH1 gene deleted, JBEI 10691; Crosses represent BY4741 with MvaES, ACLab, and the *Y. lipolitica* IDH genes expressed JBEI 10692. Strains were grown in low [(NH_4_)_2_SO_4_]

When *mvaE* and *mvaS* were expressed in a wild-type background, we observed a significant increase in both mevalonate and the downstream metabolite squalene (Fig. 4a, b). Squalene has previously been shown to accumulate in strains engineered for increased flux to the mevalonate pathway [8, 37]. Expressing ACL with *mvaE* and *mvaS* did not lead to an increase in mevalonate concentration, and led to only a ~twofold increase in squalene concentration. However, when ACL, *mvaE*, and *mvaS* were expressed in an *IDH1Δ* background, we observed significant increases in both mevalonate and squalene (137 and 445 %, respectively, increase over the strain engineered with *mvaE,* and *mvaS* expression only). These data demonstrate that while expression of the upper mevalonate pathway genes *mvaE* and *mvaS* leads to mevalonate accumulation, a substantial amount of produced mevalonate is converted to downstream products such as squalene, and may also accumulate as other downstream products (Fig. 1). Lastly, the combined strategy of ACL expression with *IDH1Δ* led to a ~threefold increase in mevalonate production over strains without these two modifications. This is consistent with our previous results, and indicates that shunting citrate from the TCA cycle via *IDH1Δ* in combination with expression of ACL is a viable strategy for increasing flux to the mevalonate pathway.

Interestingly, the best producing strain (JBEI 10691–*IDH1Δ*; ACL; *mvaES*) also exhibited substantially less growth than the *IDH1*^+^ strains (Additional file 3: Figure S2). We reasoned that this may have been caused by reduced citrate flux to the TCA cycle. We initially hypothesized that replacing *S. cerevisiae* ICDH with one from an oleaginous yeast might achieve a flux distribution between the TCA cycle and the ACL pathway that would allow for improved growth and similar or higher titers, as previous work has shown that ICDH from oleaginous yeast are responsive to the carbon/nitrogen molar ratio [43]. To test this, genes from *Y. lipolytica* with homology to *S. cerevisiaeIDH1* and *IDH2* were identified, and their functionality was confirmed through complementation of *S. cerevisiaeIDH1/2Δ* (Additional file 4: Figure S3). However, when the *Y. lipolytica* ICDH replaced *IDH1*, we observed reduced production compared with *IDH1Δ* (Fig. 4). The failure of *Y.l.IDH1/2* to improve mevalonate production is likely due to the different regulatory environment between oleaginous yeasts and *S. cerevisiae*.

### Engineering a strain for high mevalonate production

**Fig. 5:**
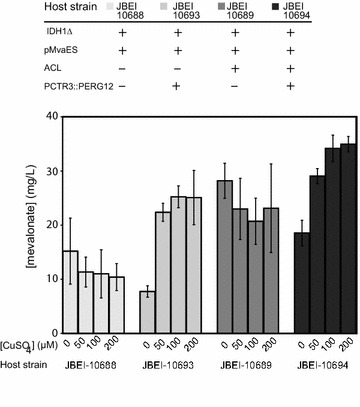
Comparison of mevalonate production levels by engineered *S. cerevisiae* strains with and without the P*CTR3*::P*ERG12* promoter replacement, and strains with and without ACL, grown in various concentrations of CuSO_4_. All strains carry *IDH1∆* and harbor the *pMvaES* plasmid. Strains were grown in low [(NH_4_)_2_SO_4_]

Having demonstrated that expressing ACL in the *IDH1Δ* background serves to increase flux to the engineered mevalonate pathway, we next focused our efforts on further increasing production of the pathway intermediate mevalonate. We first determined that mevalonate does not inhibit growth, nor is it consumed as a carbon source (Additional file 5: Figure S4 A, B). We then determined that mevalonate does not accumulate intracellularly, and is efficiently exported extracellularly (Additional file 6: Figure S5). To engineer mevalonate accumulation, *mvaE* and *mvaS* were expressed from a high copy plasmid so as to maximize flux from acetyl-CoA to mevalonate. Then, we attempted to reduce flux downstream of mevalonate by modifying the expression of mevalonate kinase, the next step in the mevalonate pathway. This activity is encoded by *ERG12*, which is essential, as flux through the mevalonate pathway is necessary for production of sterols, lipids and a host of other metabolites (Fig. 1). In order to achieve an expression level that allowed enough flux through the mevalonate pathway to meet essential requirements, while limiting accumulation of other pathway intermediates, the *ERG12* promoter was replaced with the copper-repressible *CTR3* promoter. Consistent with our previous results, expression of ACL (without the P*CTR3::*P*ERG12* modification) resulted in an approximately twofold increase in mevalonate production. When the P*CTR3::*P*ERG12* modification was introduced (without ACL expression), *ERG12* repression resulted in a twofold increase in mevalonate production. When the two modifications were combined, we observed a modest but significant increase in mevalonate production as compared to strains engineered with either of the individual modifications, ultimately leading to production of over 30 mg/liter of mevalonate (Fig. 5).

## Conclusions

In this study the yeast *S. cerevisiae* was engineered with an alternative mode of acetyl-CoA biosynthesis mediated by ACL in conjunction with a push/pull/block strategy. As a proof of principle, we demonstrate that this alternative mode of metabolism can be used as a strategy to significantly increase the yield of the acetyl-CoA derived product mevalonate. We demonstrate the basic requirements for directing flux through the ACL pathway: increased citrate supply, expression of an active ACL, increased pull towards a product of interest, and limitation of competing downstream pathways. We anticipate that this strategy can be extended to the production of a wide array of acetyl-CoA derived molecules.

## Additional files

10.1186/s12934-016-0447-1Additional plasmids used for strain and expression plasmid construction.
10.1186/s12934-016-0447-1Intracellular concentrations of a panel of key related metabolites of depicted strains grown in low nitrogen. *Oxaloacetate was not detected, as it is very labile.
10.1186/s12934-016-0447-1Growth curves of engineered strains pertaining to Fig. 4. Strains were grown in low [(NH_4_)_2_SO_4_].
10.1186/s12934-016-0447-1Growth of *S. cerevisiae* *IDH1Δ/2Δ* and *IDH1Δ/2Δ* complemented with *IDH1* and *IDH2* from *Y. lipolytica* on dextrose (fermentable) and acetate (non-fermentable).
10.1186/s12934-016-0447-1Growth curves of wild type cultures grown with various mevalonate concentrations added to media at time zero (A) and measurement of mevalonate concentrations in broth after various mevalonate concentrations added to cultures at time zero (B).
10.1186/s12934-016-0447-1Comparison of intracellular and extracellular amounts of mevalonate found in the 1 mL of culture broth. Strain shown is the *S. cerevisiae* host strain JBEI-10688 harboring the *pMvaES* plasmid.
