# Supplementary material for: ATP citrate lyase mediated cytosolic acetyl-CoA biosynthesis increases mevalonate production in Saccharomyces cerevisiae
Source: Microb Cell Fact. 2016 Mar 3;15:48. doi: 10.1186/s12934-016-0447-1 (PMC4778282; doi:10.1186/s12934-016-0447-1)
Supplement: Supplementary file 1 — 10.1186/s12934-016-0447-1Additional plasmids used for strain and expression plasmid construction. [file 12934_2016_447_MOESM1_ESM.docx]

**Table S1. Additional plasmids used for strain and expression plasmid construction**

| Plasmid | Description | Source |
| --- | --- | --- |
| JBEI-10630 | p*Ori-AmpR-5’*_IDH1_*-*P_GAL10_*-YlIDH1*-P_GAL1_*-YlIDH2*-kan*MX4*-*3’*_IDH1_ | This study |
| JBEI-10631 | p*Ori-AmpR-5’*_TRP1_*-*P_GAL10_*-EfMvaE*-P_GAL1_*-EfMvaS*-SpHIS5-*3’*_TRP1_ | This study |
| JBEI-10641 | p*Ori-AmpR-5’*_URA3_*-*P_GAL1_*-RtACL*-kan*MX4*-*3’*_URA3_ | This study |
| JBEI-10642 | p*Ori-AmpR-5’*_URA3_*-*P_GAL1_*-MmACLa*-kan*MX4*-*3’*_URA3_ | This study |
| JBEI-10643 | p*Ori-AmpR-5’*_URA3_*-*P_GAL10_*-YlACLb*-P_GAL1_*-YlACLa*-kan*MX4*-*3’*_URA3_ | This study |
| JBEI-10644 | p*Ori-AmpR-5’*_URA3_*-*P_GAL10_*-LsACLb*-P_GAL1_*-LsACLa*-kan*MX4*-*3’*_URA3_ | This study |
| JBEI-10645 | pESC*-*P_GAL10_*-YlACLb*-P_GAL1_*-YlACLa*-*LEU2d* | This study |
| JBEI-10646 | pESC*-*P_GAL10_*-LsACLb*-P_GAL1_*-LsACLa*-*LEU2d* | This study |
| JBEI-10647 | pESC*-*P_GAL10_*-EfMvaE*-P_GAL1_*-EfMvaS* -P _GAL1_*-ADS-LEU2d* | This study |
| JBEI-10648 | p*Ori-AmpR-5’*_URA3_*-*P_GAL10_*-AnACLb*-P_GAL1_*-AnACLa*-kan*MX4*-*3’*_URA3_ | This study |
| JBEI-10649 | pESC-P_GAL1_*-RtACL*-*LEU2d* | This study |
| JBEI-10651 | pESC*-*P_GAL1_*-MmACL*-*LEU2d* | This study |
| JBEI-10682 | pESC*-*P_GAL10_*-AnACLb*-P_GAL1_*-AnACLa*-P _GAL1_*-ADS-LEU2d* | This study |
