# Supplementary figures and images for: ATP citrate lyase mediated cytosolic acetyl-CoA biosynthesis increases mevalonate production in Saccharomyces cerevisiae
Source: Microb Cell Fact. 2016 Mar 3;15:48. doi: 10.1186/s12934-016-0447-1 (PMC4778282; doi:10.1186/s12934-016-0447-1)

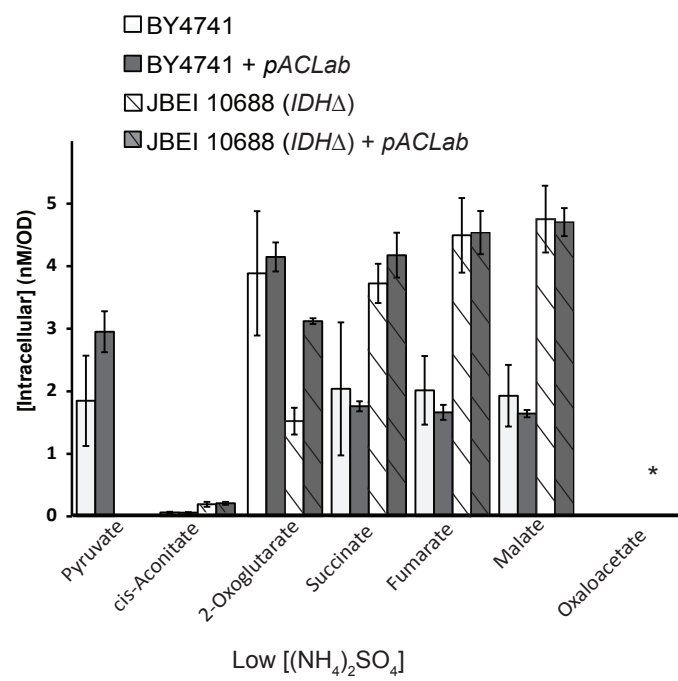

Supplement: Supplementary file 2 — 10.1186/s12934-016-0447-1Intracellular concentrations of a panel of key related metabolites of depicted strains grown in low nitrogen. *Oxaloacetate was not detected, as it is very labile. [file 12934_2016_447_MOESM2_ESM.pdf]

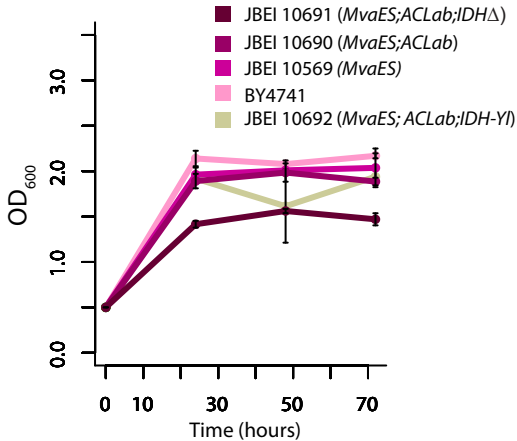

Supplement: Supplementary file 3 — 10.1186/s12934-016-0447-1Growth curves of engineered strains pertaining to Fig. 4. Strains were grown in low [(NH4)2SO4]. [file 12934_2016_447_MOESM3_ESM.pdf]

Dex -Leu

Acetate -Leu

*S.c. IDH1Δ/2Δ*

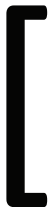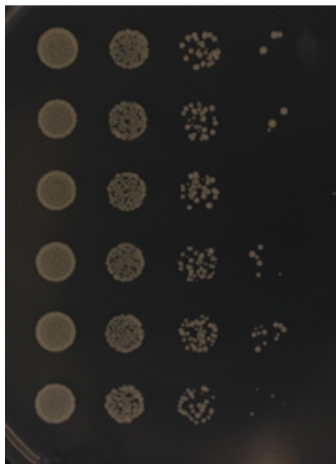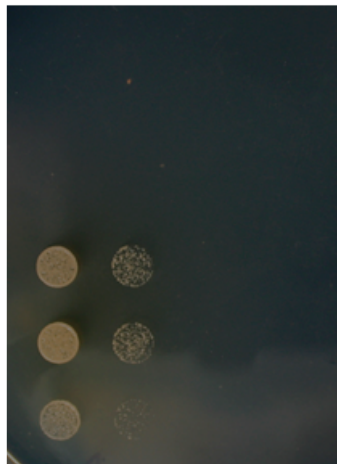

*S.c. IDH1Δ/2Δ*  
+ *Y.l. IDH1/2*

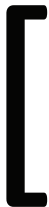

Supplement: Supplementary file 4 — 10.1186/s12934-016-0447-1Growth of S. cerevisiae IDH1Δ/2Δ and IDH1Δ/2Δ complemented with IDH1 and IDH2 from Y. lipolytica on dextrose (fermentable) and acetate (non-fermentable). [file 12934_2016_447_MOESM4_ESM.pdf]

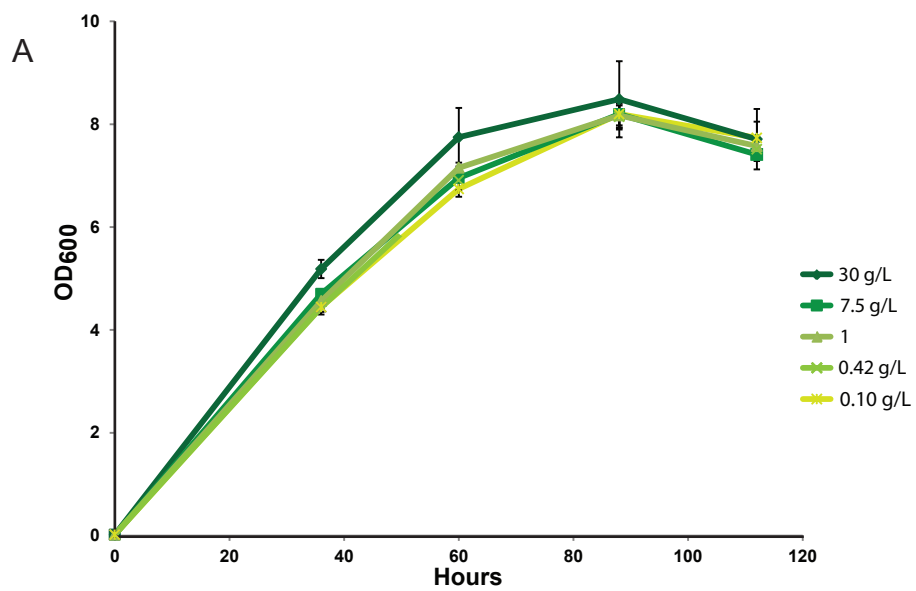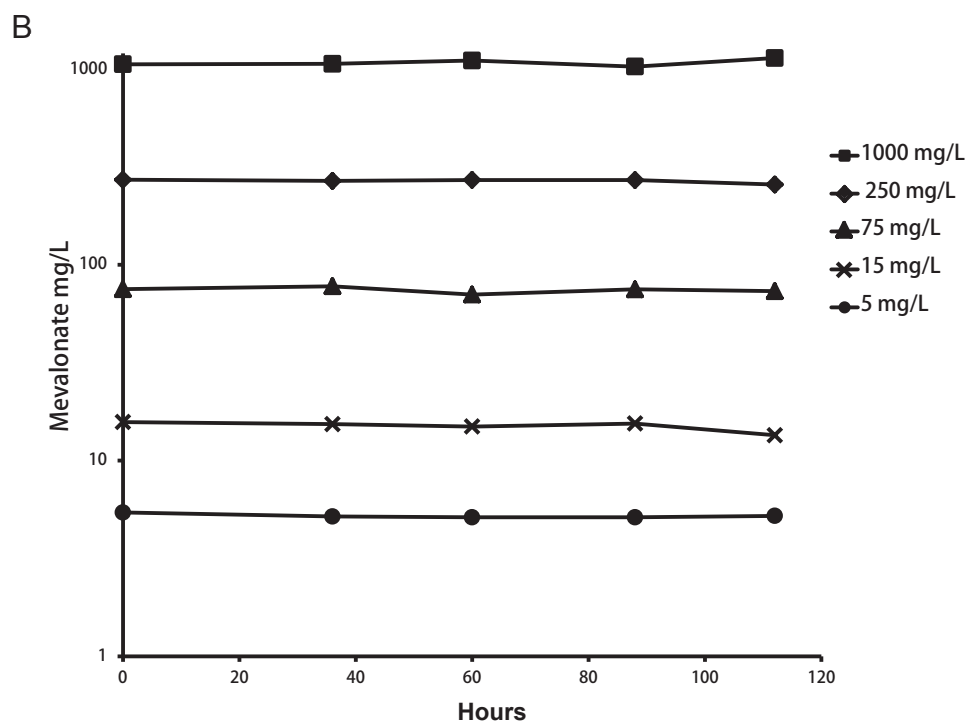

Supplement: Supplementary file 5 — 10.1186/s12934-016-0447-1Growth curves of wild type cultures grown with various mevalonate concentrations added to media at time zero (A) and measurement of mevalonate concentrations in broth after various mevalonate concentrations added to cultures at time zero (B). [file 12934_2016_447_MOESM5_ESM.pdf]

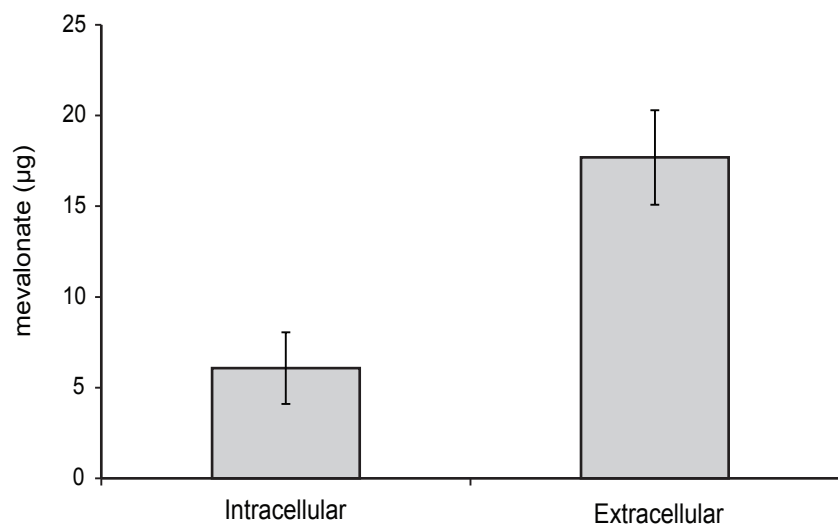

Supplement: Supplementary file 6 — 10.1186/s12934-016-0447-1Comparison of intracellular and extracellular amounts of mevalonate found in the 1 mL of culture broth. Strain shown is the S. cerevisiae host strain JBEI-10688 harboring the pMvaES plasmid. [file 12934_2016_447_MOESM6_ESM.pdf]
